# Distribution of visual and saccade related information in the monkey inferior colliculus

**DOI:** 10.3389/fncir.2012.00061

**Published:** 2012-09-05

**Authors:** David A. Bulkin, Jennifer M. Groh

**Affiliations:** ^1^Department of Psychology, Cornell UniversityIthaca, NY, USA; ^2^Department of Neurobiology, Duke UniversityDurham, NC, USA; ^3^Department of Psychology and Neuroscience, Duke UniversityDurham, NC, USA; ^4^Center for Cognitive Neuroscience, Duke UniversityDurham, NC, USA

**Keywords:** multisensory, auditory, vision, cross-modal

## Abstract

The inferior colliculus (IC) is an essential stop early in the ascending auditory pathway. Though normally thought of as a predominantly auditory structure, recent work has uncovered a variety of non-auditory influences on firing rate in the IC. Here, we map the location within the IC of neurons that respond to the onset of a fixation-guiding visual stimulus. Visual/visuomotor associated activity was found throughout the IC (overall, 84 of 199 sites tested or 42%), but with a far reduced prevalence and strength along recording penetrations passing through the tonotopically organized region of the IC, putatively the central nucleus (11 of 42 sites tested, or 26%). These results suggest that visual information has only a weak effect on early auditory processing in core regions, but more strongly targets the modulatory shell regions of the IC.

## Introduction

Recent work has implicated the inferior colliculus (IC) in audio-visual integration. IC neurons can show different responses to auditory stimuli when presented with concurrent visual stimuli, and can even exhibit overt responses in the absence of a sound (Mascetti and Strozzi, 1988; Gutfreund et al., 2002; Porter et al., 2007; Bergan and Knudsen, 2009) (for review, see Gruters and Groh, this issue). The IC is a critical site of convergence along the ascending auditory pathway: virtually all auditory signals from the brainstem must stop in the IC before reaching thalamus and eventually cortex (Aitkin and Phillips, 1984; Winer and Schreiner, 2005). Thus, visual effects in the IC have the potential to exert a powerful influence on subsequent auditory processing. However, the subnuclei of the IC have dramatically different connectivity patterns. In order to interpret the implications of visually sensitive neurons in the IC it is essential to localize them within the structure.

Recently, our group demonstrated visually sensitive neurons in the macaque IC during a visual fixation task (Porter et al., 2007). A large proportion of sampled units (64%) showed changes in activity that were time-locked to either the onset of a visual fixation cue and/or eye movements that followed. However, because recording location was not systematically varied, it has remained unclear whether these responses are found throughout the IC or are confined to a subregion. In owls, visual modulation of activity has been localized within the lateral nucleus (Gutfreund et al., 2002; Bergan and Knudsen, 2009), but comparable data for other species are sparse and the possibility remains that other regions also show visual sensitivity.

Identifying the precise location of recordings by combining physiology and histology is particularly challenging in large animals such as, macaques. Physiological data are collected over several months, with many repeated electrode penetrations, and individual animals often participate in several experiments. Thus, by the time anatomical data are collected and analyzed, the location at which individual units were recorded is unclear. If only a few histologically-confirmed recording sites can be established for each animal, the time, effort, and cost involved in accumulating a sufficient sample is prohibitive.

One alternative to direct anatomical methods is to utilize known topographic patterns to form a functional map. This approach provides an indirect estimate of location, but allows measures that are centered on the recording electrode specific to each recording penetration. In this manner, the location of neurons possessing a particular functional feature (i.e., sensitivity to visual and visuomotor events) can be assessed using a previously ascertained organization (i.e., functional auditory topography).

We recently defined such a functional map by systematically sampling auditory responses in macaques (Bulkin and Groh, 2011). We measured multiunit neuronal activity throughout the midbrain of six macaques while presenting sounds, allowing delineation of several subregions of the macaque IC consisting of an area showing a tonotopic organization, a surrounding area with neurons tuned for low frequency sounds (tuned area), and a peripheral area in which neurons were either not tuned for tone frequency or not responsive to tones (untuned area). Using this functional map, we presently identify the locations of visually responsive neurons.

Overall, 84 of 199 (~42%) sampled locations showed a change in firing rate following the onset of the fixation stimulus. Responses were most common and most powerful in recordings taken along untuned penetrations, located chiefly in the rostral portion of our recording zone. In penetrations passing through the low frequency tuned, non-tonotopically organized, region of the IC, fewer (and weaker) responses were found. Along tonotopic penetrations, we found only subtle (though significant) effects of visual stimulus presentations. Despite the lack of robust spiking responses in the tonotopically organized region of the IC, evoked potentials from this area were on par with recordings from neighboring subregions.

These results exhibit similarities and differences compared to our recent findings concerning the distribution of eye-position sensitivity in the IC (Bulkin and Groh, 2012). Neurons sensitive to the orientation of the eyes were most prevalent in recordings in tuned and untuned penetrations, like we report here for visual sensitivity. However, the amplitude of eye position modulation was similar throughout all regions of the IC, whereas we report here that modulation of neuronal activity in response to visual stimuli was much weaker along tonotopic penetrations, putatively the central nucleus of the IC, than in other areas. Taken together, the pattern of overt visual responses and eye position sensitivity supports the view that these multisensory influences are generally more powerful in the tuned and untuned areas, where neuronal activity was strongly related to both the position of the eyes and presentation of visual stimuli, than in the tonotopic area. The dissociation of these two effects in the tonotopically organized IC suggests these two signals may play somewhat different roles in the core ascending auditory pathway.

## Materials and methods

### Surgical preparation, recording procedures, and inclusion criteria

Three male and three female rhesus monkeys participated in the experiments. All procedures were approved by the Institutional Animal Care and Use Committees at Dartmouth College and Duke University, and were conducted in accordance with the principles of laboratory animal care of the National Institutes of Health (publication 86-23, revised 1985). Surgical procedures were performed using isoflurane anesthesia and aseptic techniques as well as postoperative analgesia. The monkeys underwent an initial surgery to implant a head post for restraining the head and a scleral eye coil for monitoring eye position (Robinson, 1963; Judge et al., 1980). After recovery, an additional surgery was performed to make a craniotomy and to implant a recording cylinder positioned to allow electrodes to approach the left IC at an angle approximately 30° from vertical in the coronal plane. The chamber contained a fixed grid of holes (Crist Instruments, Gaithersburg, MD) aligned such that electrode penetrations could be made in 1 mm increments in the anterior/posterior and (tilted) medial/lateral dimensions.

Recordings were made using tungsten microelectrodes (1–3 MΩ; FHC Inc., Bowdoin, ME). Multiunit clusters were selected using a window discriminator (Monkeys A,W: Plexon Inc., Dallas, TX; Monkeys E,M: Bak Electronics, Germantown, MD) and times of action potentials were stored for off-line analysis. A separate local field potential (LFP) signal (band pass filtered between 0.7 and 300 Hz) was collected and digitized (sampling rate 20 KHz). The location of the IC was determined using an anatomical MRI scan in which the recording chamber and plastic grid could be visualized, and was verified using physiological responses. Using MRI, we estimated all of the borders of the IC except on the rostral aspect, where a clear definition was not visible. This study was part of a larger mapping study in which we sampled neuronal activity throughout the region (in 1 mm increments in the transverse plane and 0.5 mm increments along the depth axis of our recording penetrations) to form a functional map of the region (Bulkin and Groh, 2011). Recordings to assess visual sensitivity were taken at a subset of locations, limited by the behavioral performance of the monkeys, which was required for the visual assessment but not for auditory assessment. Recording sessions began and ended at depths above and below the putative IC to ensure that the entire structure was covered. Analysis of visual response data was limited to locations within the borders measured by MRI scans ±1.5 mm, and meeting auditory response criteria. Sites were included if responses to sounds exceeded 3 SDs of baseline for 10 ms consecutively in a 50 ms window following auditory stimulus onset, and at least three sites within a penetration had to show such responses.

We tested a subset of sites, notably those in the rostral-most penetrations in monkey A, with microstimulation to rule out that they were in the superior colliculus (SC). The SC is an oculomotor structure rostral and dorsal to the IC, and it exhibits auditory responsiveness when animals are engaged in auditory saccade tasks (Jay and Sparks, 1984; Populin et al., 2004). Saccades can be elicited by stimulation in the SC (Robinson, 1972) but are not expected to be elicited from the IC. The absence of stimulation-evoked saccades can therefore help confirm that the MRI-based and physiological inclusion criteria described above are sufficient to limit auditory-responsive sites to those in the IC. Saccades were only elicited at 2 of 51 tested sites included in the auditory data set, confirming the adequacy of anatomical and physiological criteria. Neither of these sites was probed for visual responses, so they were not analyzed further for the present study. In more dorsal, also non-included locations, saccades were frequently observed following stimulation, with a predictable topographic organization of evoked saccade vectors. Such sites were probably in the SC.

### Stimulus presentation and behavioral task

Experiments were conducted in complete darkness in a single-walled sound isolation booth (International Acoustics Company, New York, NY). Echo-absorbent material lined the walls and ceiling (3 inches, Sonex Painted One acoustic foam), as well as the floor (carpet). The head of the monkey was restrained throughout the experiment. Visual stimuli consisted of light emitting diodes (LEDs; luminance ~26.4 cd/m^2^) presented from an array located ~57 inches in front of the head.

In designing a task for this study, we were motivated by several considerations. The previous finding of visual responses in the monkey IC was a somewhat opportunistic one: visual responses were observed in conjunction with the onset of, or saccade to, a visual fixation target in a task in which visual fixation was followed by sound presentations (Porter et al., 2007). We wanted the task in the current study to also involve a visual fixation stimulus, and a similar time course, so that we would be able to make direct comparisons with the previous study. The task used previously included the presentation of a sound. This auditory stimulus was presented after the periods probed for visual-related responses, but we could not rule out the possibility that anticipation of the sound contributed to visual responsiveness. Here, we wanted to eliminate any chance that a temporally-linked auditory stimulus might be connected to the observed visual effects. Thus, in the present experiments we used tasks that contained no auditory stimuli.

**Figure 1:**
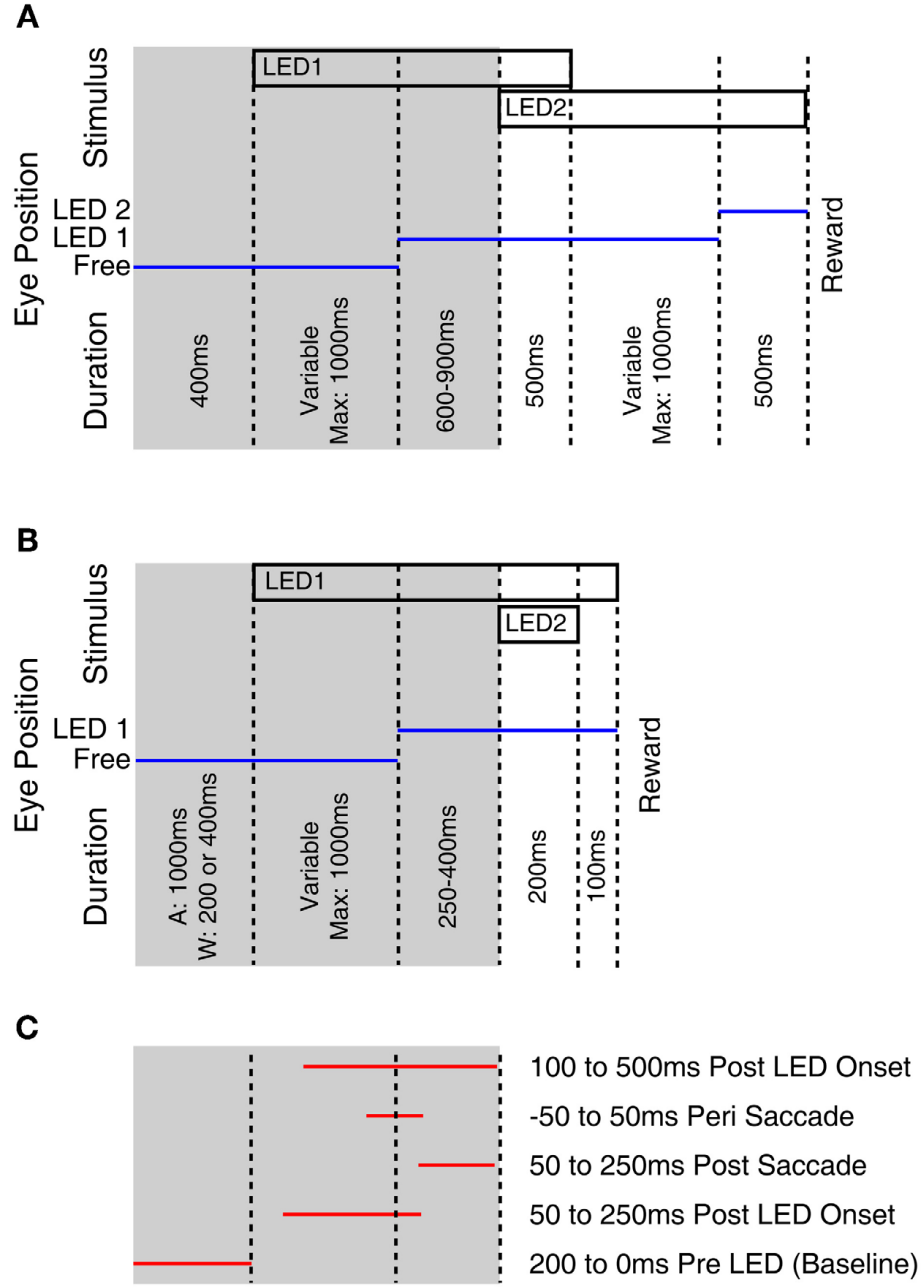
**Fixation tasks used to probe for visual responses.** This figure illustrates the behavioral tasks used to probe for visual stimuli. Monkeys E and M performed a multiple fixation task **(A)** whereas monkeys A and W held fixation while ignoring a probe stimulus **(B)**. Temporal intervals tested for visual modulation of activity **(C)** used only the first part of the trials, that was common across monkeys. The tested epochs indicated in **(C)** are not drawn to scale as trials involved variable time intervals.

Monkeys were trained using operant conditioning to fixate visual stimuli for a fluid reward. At each recording site approximately 200 rewarded trials were collected. Two task designs were used and are depicted in Figure 1. Both tasks began with the fixation of an LED presented at eye level. Subsequently an additional “probe” LED was briefly flashed at 1 of 12 locations. Monkeys E and M were trained to make an additional saccade to the probe stimulus following the offset of the initial fixation LED (Figure 1A). Monkeys A and W were trained to ignore the probe LED and maintain fixation on the initial LED (Figure 1B). To allow comparison with our previous study and across monkeys within this study, all of the neurophysiological responses reported in this paper derive from epochs associated with the onset of this fixation target and its associated saccade (the shaded regions in Figure 1), i.e., when the task was similar across subjects and studies. If the monkey failed to fixate the appropriate target, or broke fixation, the trial was unrewarded and not included in the analysis. A variable interval ranging from 1 to 2 s followed the reward or error during which no stimuli were presented.

Acoustic stimuli used to ascertain tuning characteristics were presented in separate blocks at the sites probed for visual responses, as well as at numerous sites not tested for visual responsiveness. Auditory stimuli were presented on unrewarded trials with no other stimuli or performance requirements, and consisted of tones of 16 frequencies ranging from 0.4 to 12 kHz (approximately ¼ octave increments), as well as broadband noise (spectrum ranging from 0.5 to 18 kHz). Sounds were generally presented using loudspeakers (Audax Model TWO25V2 or Bose Acoustimas cube speakers) located 90° contralateral to the recording chamber and 57 in. from the subject's head. In all monkeys sounds were presented at 50 dB SPL, calibrated to within 1 dB of the target amplitude using a sound meter (Brüel & Kjær, model 2237 with model 4137 condenser microphone; A-weighted) placed at the position that the monkey's head would occupy in the experiment. The neural responses to sounds were used to classify recordings in a map of IC auditory topography.

### Data analysis

Data were analyzed off-line to determine which sites along a penetration showed auditory responses. Auditory penetrations (showing three or more responsive sites) were classified as tonotopic if they showed a systematic progression of increasing tuned frequency with depth, tuned if three or more recordings within a penetration showed frequency tuning (but no systematic progression of tuned frequency), or untuned if the sites either did not respond to tones or did but responded similarly for all tone frequencies. The methods for the auditory response analysis have been described previously (Bulkin and Groh, 2011).

To determine if visual stimuli elicited a response, we compared firing rates during a baseline period (200 ms preceding stimulus onset) to the firing rate during four response epochs: 50–250 ms following the LED illumination, 50–250 ms following the saccade to the LED, 100 ms centered on the saccade, and 100–500 ms following onset of the LED (Figure 1C, Table 2). We used a two-tailed paired *t*-test to look for significant changes in activity, with a Bonferroni corrected criterion alpha value (i.e., *p* < 0.0127). The rationale for probing for responses in these epochs was to detect sites with different response properties. For example, sites with receptive fields in different locations might exhibit responses during different time periods, based on the eye movements required to perform the task. Sites with extra-foveal receptive fields might respond during the initial appearance of the LED if it happened to lie within that receptive field. Sites with foveal receptive fields would be more likely to show a response at the completion of a saccade to the LED, which would bring the LED onto the fovea. Sites with activity coupled to the saccade would show activity during the time window centered on the saccade. And we have previously observed some sites with slowly increasing activity following the onset of the stimulus (Porter et al., 2007).

Occasionally, electrical noise generated by the LED apparatus influenced recording equipment and generated a spurious response. Artifacts were readily detected as increases in activity following the onset of the LED with no latency. These sites were removed from analysis by probing for increases in activity exceeding two standard deviations of baseline firing during the first 20 ms of following stimulus onset. Seventeen recordings were excluded from analysis due to too-short latency changes in firing rate.

To investigate spatial sensitivity to visual stimuli, we fit a two-dimensional Gaussian surface to the response (in each of the windows used for response detection described above) as a function of the horizontal and vertical location of the retinal or eye-centered stimulus location.

$$f\left(x,y\right)=\text{C}+{\text{Ae}}^{-\left(\frac{{\left(x-\mu_{x}\right)}^{2}}{2\sigma_{x}^{2}}+\frac{{\left(y-\mu_{y}\right)}^{2}}{2\sigma_{y}^{2}}\right)}$$

*f*(*x*, *y*) is the estimated firing rate response at eye-centered horizontal (*x*) and vertical (*y*) locations of the stimulus at onset. As the eyes were freely moving before the initial fixation cue, there was spontaneous variation in the retinal position of the stimulus. Estimates of μ_*x*_ and μ_*y*_ define the center of the receptive field, and σ_*x*_ and σ_*y*_ the spread of the responsive area in the horizontal and vertical dimensions. The amplitude, A, describes the ratio between responsive and unresponsive regions. We allowed both positive and negative amplitude Gaussian fits, meaning that the center of the surface could represent a peak or trough. To compare receptive fields across the population we calculated the diameters of an ellipse made by the Gaussian at half the height of the peak with respect to the offset:

$$\begin{array}{l} {\text{FWHM}}_{x}=\sigma_{x}\cdot 2\sqrt{2\text{ln 2}}\\ {\text{FWHM}}_{y}=\sigma_{y}\cdot 2\sqrt{2\text{ln 2}} \end{array}$$

## Results

We tested for visual responses in the activity of multiunit clusters distributed throughout the IC of four rhesus macaques. A total of 84 of 199 recording sites showed a response to the presentation and fixation of an LED in at least one of four tested epochs surrounding the events (Tables 1, 2). Our most extensive sample came from monkey A due to improvements in the techniques we used to target potential recording sites.

**Table 1:** **Proportion of visual/visuomotor sensitive sites in functionally defined locations**.

	**Monkey**				
	**A**	**W**	**E**	**M**	**Total**
Tonotopic	6/27 (22.2)	5/13 (38.5)	0/0	0/2 (0)	11/42 (26.2)
Tuned	28/82 (34.1)	2/8 (25)	3/14 (21.4)	2/6 (33.3)	35/110 (31.8)
Untuned	34/43 (79.1)	3/3 (100)	1/1 (100)	0/0	38/47 (80.9)
Total	68/152 (44.7)	10/24 (41.7)	4/15 (26.7)	2/8 (25)	84/199 (42.2)

**Table 2:** **Proportion of visual/visuomotor sensitive sites in functionally defined locations by response epoch**.

	**Epoch 1 50–250 ms LED onset (%)**	**Epoch 2 50–250 ms saccade (%)**	**Epoch 3 100–500 ms LED onset (%)**	**Epoch 4 −50 to 50 ms saccade (%)**	**Group 1 Epochs 1 or 2 or 3 (%)**	**Group 2 Group 1, not epoch 4 (%)**	**Group 3 Epoch 4. not group 1 (%)**
Tonotopic (*n* = 42)	6 (14.3)	8 (19)	8 (19)	7 (16.7)	9 (21.4)	4 (1)	0 (0)
Tuned (*n* = 110)	22 (20)	17 (15.5)	22 (20)	17 (15.5)	33 (30)	18 (16.4)	2 (0.2)
Untuned (*n* = 47)	32 (68.1)	25 (53.2)	32 (68.1)	34 (72.3)	36 (76.6)	4 (0.9)	2 (0.4)
Total (*n* = 199)	60 (30.2)	50 (25.1)	62 (31.2)	58 (29.1)	78 (39.2)	26 (13.1)	4 (0.2)

**Figure 2:**
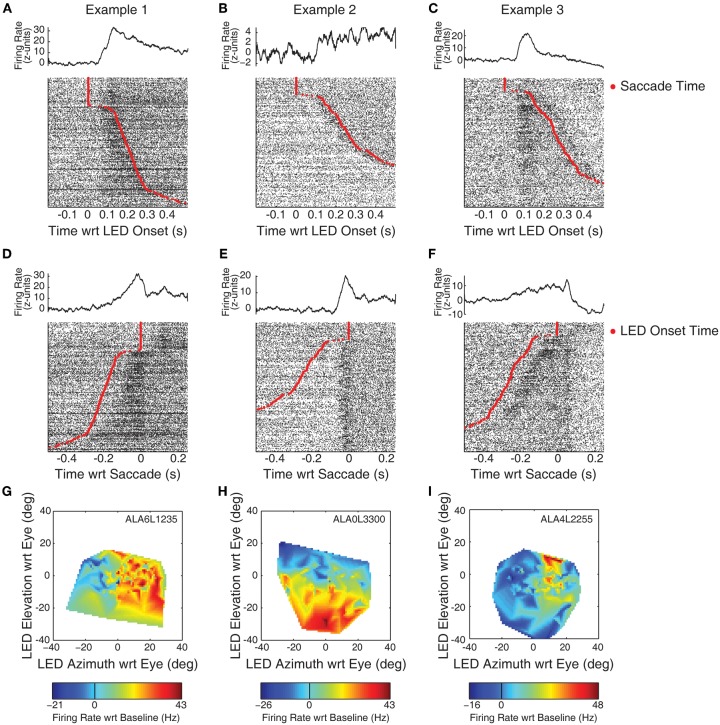
**Example recordings with visual and visuomotor related activity.** Neural spiking data is plotted for each of three example recording sites in the IC. Panels **(A–C)** display PSTHs and rasters aligned on the onset of the LED. The time of the saccade is indicated by red points in the rasters. An increase in firing rate, time-locked to the onset of the LED, can be observed in panels **(A)** and **(C)**. Panels **(D–F)** display the same data, but aligned instead on the time at which the monkey's eyes entered the criterion fixation window (i.e., on the red points in **A**–**C**). Here, a seemingly motor-related response can be seen in panel **(E)**. Panels **(G–I)** indicate the spatial tuning properties of the responses using spontaneous changes in eye orientation at the time of LED onset. The ordinate and abcissa indicate the location of the fixation stimulus in eye-centered co-ordinates. Color tracks the firing rate (subtracting baseline), warmer colors indicate increases in firing rate, while cooler colors indicate decreases.

Figure 2 displays example response patterns of multiunit clusters from three recording locations. IC cells show a mix of visual and visuomotor activity (Porter et al., 2007). Objectively classifying responses as related to visual or motor events is impossible in a simple fixation task as the onset of the LED is temporally linked to the onset of the saccade. Further confusing the issue, IC visual responses show spatial sensitivity, so a response temporally aligned with saccades may indicate either motor related activity (if it occurs prior to the saccade) or the repositioning of the visual stimulus on the retina (if it occurs following the saccade). Nonetheless, conjectures about the event best linked to responses can be garnered by investigating raster plots sorted on the time of the saccade. Figures 2A–C shows histograms and raster plots aligned on the onset of the LED. Rasters have been sorted on reaction time such that the slowest (i.e., longest duration between LED onset and saccade) responses are shown at the bottom of the panels. The red trace indicates the time the eyes entered the criterion target fixation window. At the top of the raster a number of trials are seen with no response latency; in these trials the monkey's eyes were directed at a point within the criterion window when the LED came on (though a small saccade within the window may have followed).

The examples in this figure show the range of different kinds of response patterns observed in our data. The increase in firing rate in Example 1 (panel A) appears time locked to the onset of the LED. The activity increased following the onset of the LED, and then decreased following the saccade. Whether this subsequent decrease is directly related to the saccade, or to the new position of the stimulus on the retina following the saccade (i.e., the fovea) is unclear. Example 2 (panel B) shows an increase around the time of the saccade. This increase is much clearer when the same data are aligned on the time the subject's eyes entered the fixation window (panel E). The increase in activity began immediately preceding the saccade, clearly distinguishable from a visual-related response. Nonetheless, the activity remains above baseline following the saccade, perhaps indicating the presence of a foveal receptive field. Example 3 shows increases in firing rate following both the onset of the LED and the fixation response. In both stimulus-aligned (Figure 2C) and response-aligned plots (Figure 2F), a transient increase in activity is seen following both the onset of the LED and the saccade.

Table 2 provides the results according to the epochs that indicated significant effects. Generally, sites that showed an increase in activity potentially corresponding to visual-related activity (epochs 1, 2, and 3) also showed a response in the saccade related epoch (epoch 4). (Again, note that although epoch 2 is aligned on the time of the saccade, activity during this temporal window would correspond to a visual effect, with the stimulus occupying a new location on the retina following the saccade.) Because epoch 4 overlaps with epochs 1 and 3, the data cannot unambiguously distinguish visual from motor responses. Nonetheless, virtually all sites marked as responsive in epoch 4 were also marked in one of the other epochs, suggesting a low incidence of purely motor related activity. These results generally resemble our previous findings at the single-unit level (Porter et al., 2007).

The spatial sensitivity of responses was analyzed by relating activity to the location of the LED with respect to the direction of gaze when it was illuminated. Figures 2G–I show estimates of spatial tuning for the three examples. Negative numbers on the x-axis indicate locations in the ipsilateral hemifield; negative numbers on the y axis indicate locations below fixation. Color indicates firing rate, from the epoch that best captured the response (G,I: 50–250 ms following LED onset; H: a 100 ms window centered on the saccade). Warmer colors, indicating increases in firing rate relative to baseline, are seen on the right of panel G: this site showed the largest responses to locations in the contralateral hemifield. Example 2 showed a receptive field in the lower half of the plot, this site responded best when stimuli were below spontaneous pre-trial fixation. Example 3 showed a smaller receptive field, with maximal responses when the LED was in the contralateral hemifield and near or above spontaneous pre-trial eye orientation. A similar plot based on responses in a peri-saccade window was virtually identical (data not shown).

### Anatomical distribution of visually evoked changes within the IC

**Figure 3:**
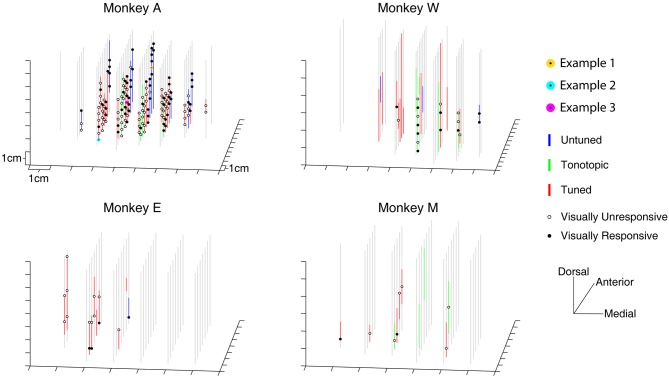
**Three dimensional map of locations of tested and responsive recording sites.** The location of visually sampled sites are portrayed in a three dimensional representation. Auditory responsive regions are indicated with colored lines untuned penetrations: blue lines; tonotopic penetrations: green lines; tuned penetrations: red lines; (Bulkin and Groh, 2011). Sites that were tested for visual responses, but did not show a significant change in firing rate are indicated with open circles, responsive locations are indicated with filled circles. The locations of the recording sites used for examples in Figure 2 are marked with yellow, cyan, and magenta circles (all were taken from Monkey A).

Visually responsive recording sites were not restricted to a small region within the IC. Figure 3 presents a three-dimensional reconstruction of recording penetrations. Grey vertical lines indicate the trajectory of recordings, which traversed through the midbrain along a dorsolateral to ventromedial course (see Materials and Methods). Along the penetrations, regions showing auditory responses are indicated by colored lines according to the categorical assignment of the penetration's auditory response (Bulkin and Groh, 2011). The locations of visually probed sites are indicated with black circles along the lines, filled markers show a change in firing rate in any of the temporal epochs tested. On occasion, the same location was tested on multiple penetrations. In these cases, a location is marked as visually responsive if any of the recordings showed a response. Responsive sites are found throughout the probed region in monkey A, and no completely unresponsive region is seen. As illustrated in Table 2, this was true for each of the different response epochs that we tested. These data indicate that modulation of activity following presentation of visual stimuli is not limited to a small subregion of the IC; rather it spanned the extent of our recordings.

**Figure 4:**
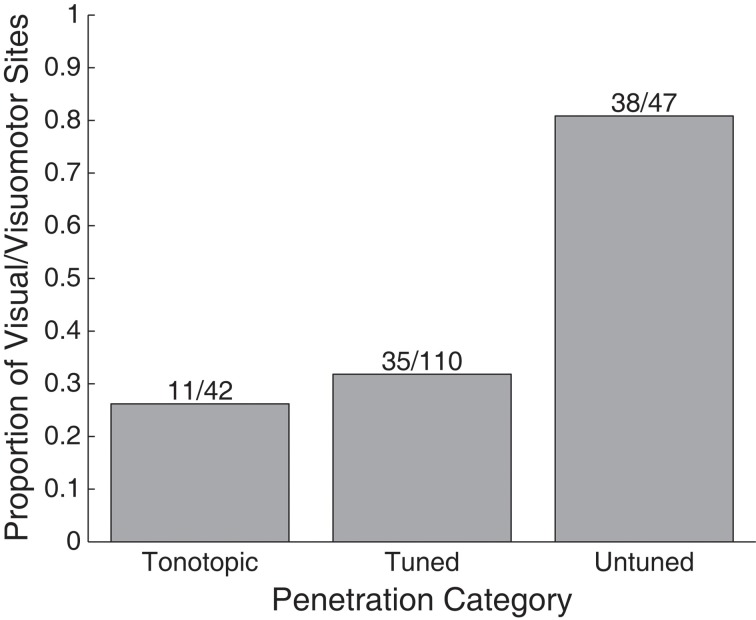
**Proportion of visually responsive recordings in tonotopic, tuned, and untuned recording penetrations.** The height of bars indicates the proportion of sites showing visual sensitivity in each auditory class of recording penetration. Untuned penetrations contained the largest proportion of sites, tuned, and tonotopic penetrations contained far fewer.

A greater proportion of visually sensitive sites are evident along the peripheral, untuned penetrations in Figure 3. Indeed, visual sensitivity was observed at the majority of sites in untuned penetrations, largely found along rostral penetrations in monkey A (Figure 4). Importantly, note that these recordings were not taken from the nearby SC. We confirmed the location of the SC was dorsal to our most rostral recordings using microstimulation to probe for evoked saccades (see Materials and Methods for details).

**Figure 5:**
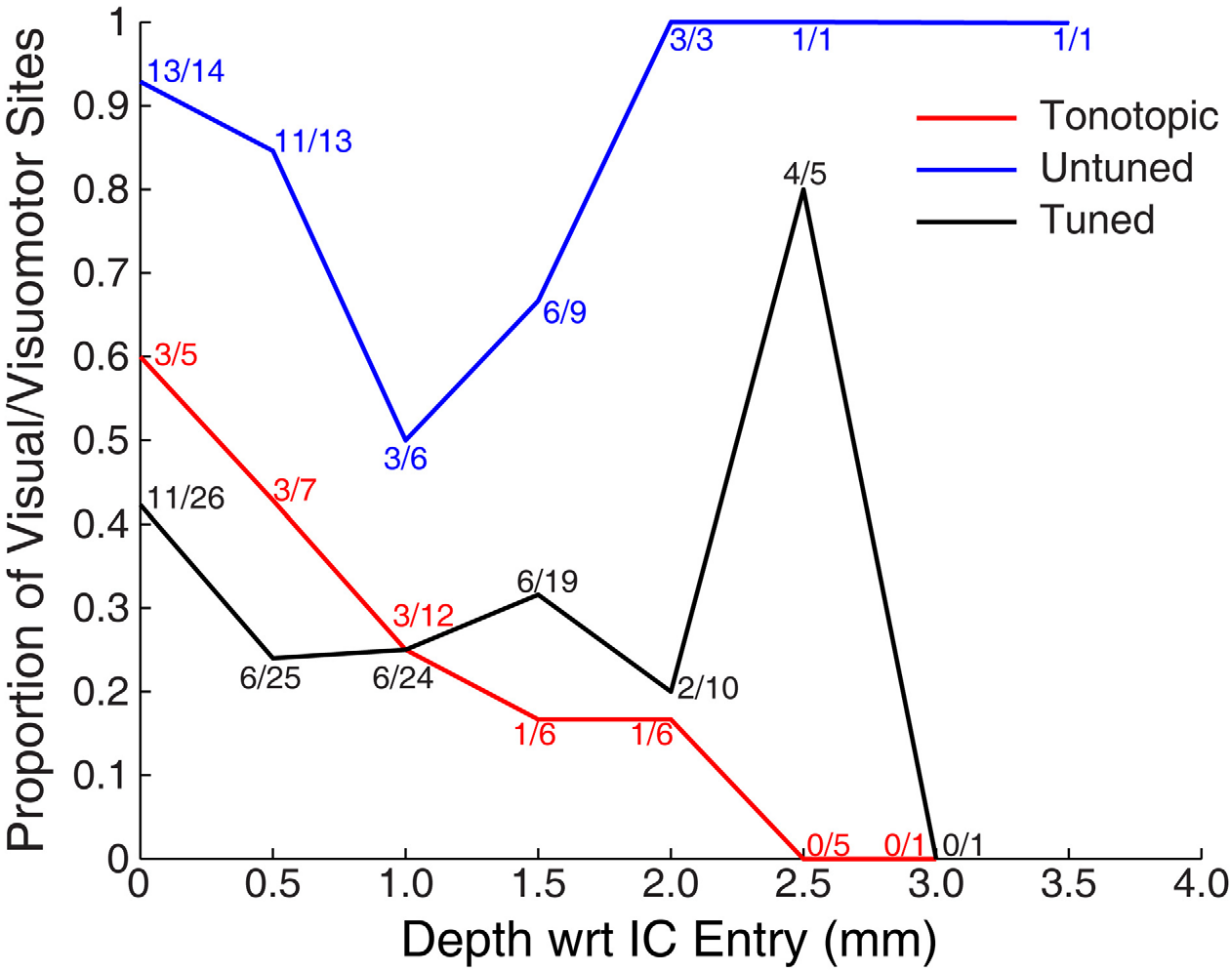
**Proportion of visually responsive recordings across recording depth.** Data were aligned on the entry into the IC (as measured by the first auditory response in a penetration, i.e., the top of the colored lines in Figure 3). Points indicate the proportion of visually sensitive sites at each relative depth, and are marked with the number of responsive and tested sites. Recordings were less common at deeper locations, in part because of the somewhat spherical shape of the IC, and in part because monkeys reached satiety over the course of early recordings.

Figure 5 shows the proportion of responsive sites as a function of recording depth for each of the penetration categories. Penetrations were aligned based on the first identified auditory responsive site (i.e., the entry into the IC). The figure provides some indication that deeper recordings within tonotopic penetrations were less likely to exhibit visual responses. However, this result should be interpreted with caution: due to variance in the length of penetrations and the alignment of the earliest sites, deep locations were somewhat undersampled. A total of 5 out of 30 sites located ≥1 mm along tonotopic penetrations showed visual responsiveness (16.7%). Although small, this proportion significantly exceeds the proportion expected by chance alone of 5% (binomial *p* < 0.05). Although not definitive, this suggests that visual responses within tonotopic penetrations are not limited to IC shell regions that the electrode passed through en route to the central nucleus.

**Figure 6:**
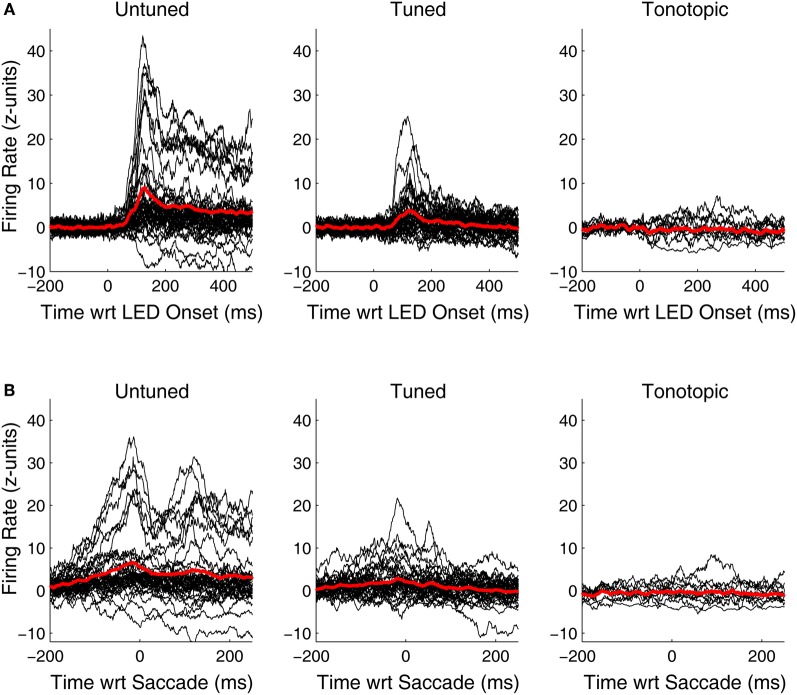
**Peri-stimulus and peri-saccade spiking histograms for visually responsive recordings in tonotopic, tuned, and untuned penetrations.** Traces of the firing rate over time for all visually sensitive sites, aligned on the onset of the LED **(A)** and the saccade **(B)** are shown in black. Spiking information was binned in 1 ms intervals, smoothed with a 20 ms moving average, and normalized in standard units of baseline (i.e., z-scores). Averages of all significantly sensitive sites (red) are overlaid. In addition to being more common, visual responses along penetrations that showed little to no frequency tuning in the auditory domain, were generally more vigorous than others. Penetrations classified as tonotopic showed only subtle changes in firing rate following presentation of a visual stimulus.

Although, visual-related signals were present and statistically significant in all three functionally defined regions, the vigor and time course of the responses differed. Figure 6 shows PSTHs for the individual responsive sites (black traces) as well as average population PSTHs (red traces) for each of the penetration categories. At untuned sites, visual responses are robust and strongly locked to the onset of the visual stimulus. At tuned sites, the responses are weaker, but some are still quite powerful and clearly locked to the visual stimulus onset. However, the pattern at tonotopic sites is quite different: the PSTHs deviate from baseline (and it is therefore clear why they are categorized as responsive) but they are weaker, and do not show the kind of crisp coupling to the LED or the saccade evident in at least at some of the sites for the other two categories. In addition, the responses appear to be equally likely to be inhibitory as excitatory.

**Figure 7:**
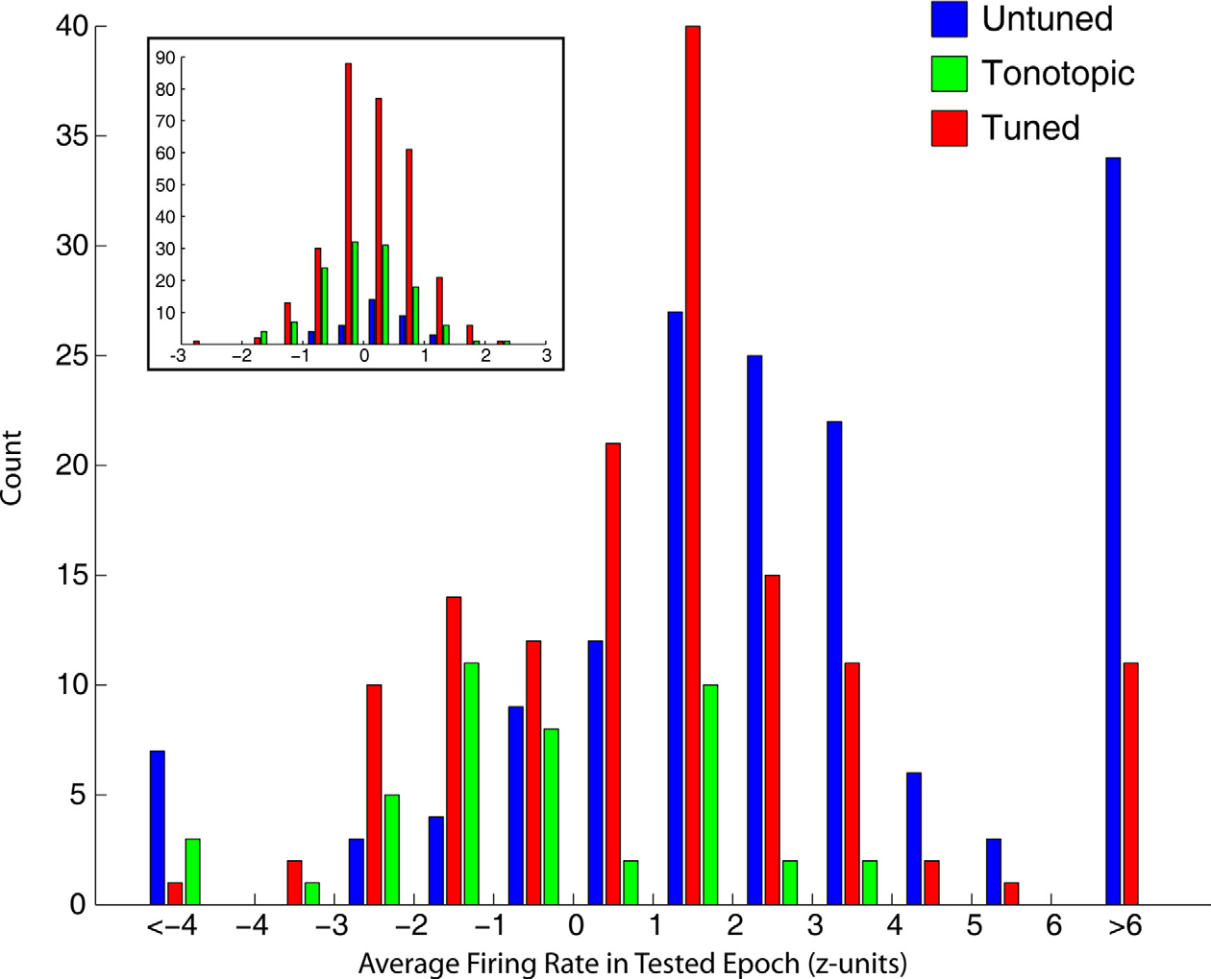
**Strength of visual responses in tonotopic, tuned, and untuned recording penetrations.** For each of the four epochs tested for visual responses, we computed the average firing rate (normalized in standard deviation units of baseline). This histogram shows the distribution of firing rates across all temporal windows in visually responsive recordings, for each of the auditory penetration categories. Visually responsive sites along untuned (blue) and tuned (red) recording penetrations showed responses of greater magnitude, and were typically excitatory. In contrast, tonotopic penetrations (green) contained sites that showed only small changes in firing rate, and were frequently inhibitory. The inset shows histograms of sites not categorized as visually sensitive, firing rate in sites marked as unresponsive was normally distributed around baseline.

The tendency of tonotopic sites to show much weaker visual-related signals is captured differently in Figure 7, which shows the distribution of average normalized (units of standard deviation of baseline) firing rate in the epochs used to mark visual responses. Visually sensitive sites in tuned and untuned penetrations generally showed excitatory responses, with untuned penetrations showing larger increases in firing rate. Visual responses in tonotopic penetrations, when found, were much smaller and frequently showed an inhibitory effect. The inset shows the distribution of firing rates for sites not marked as significantly responsive, these distributions were highly similar across categories.

**Figure 8:**
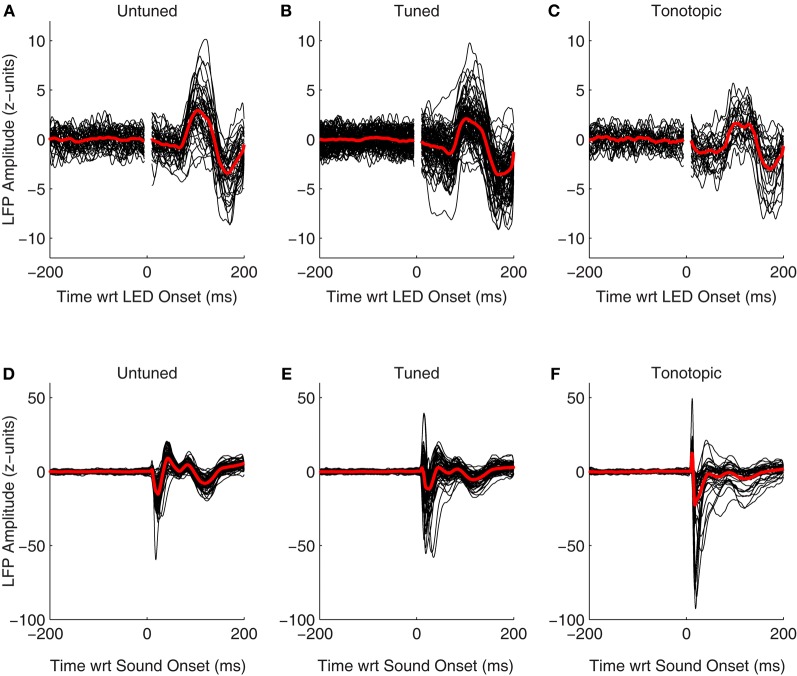
**Evoked visual and auditory potentials.** The normalized local field potentials for each site, including both those that showed significant spiking responses as well as those that did not, is plotted aligned on onset of the visual **(A–C)** and auditory stimuli (**D–F**; collected from separate blocks, see Materials and Methods). For display, traces were smoothed by convolving the raw data with a 10 ms Gaussian kernel normalized to sum to 1. The red traces indicate the mean of the responses, means were taken from the unsmoothed data.

LFPs also showed clear changes following onset of the visual stimulus. Figures 8A–C shows normalized LFPs aligned to the onset of the LED for all recorded sites in Monkey A (including both those that showed a significant spiking response as well as those that did not). Interestingly, despite the clear differences seen between spiking responses in each of the functionally defined subregions (Figures 7, 8), LFPs were quite similar. It is unclear whether changes in evoked potentials recorded are truly indicative of dendritic synaptic activity within each of subregions, or if they are merely volume-conducted changes from nearby regions. Clear differences are seen between LFPs following presentation of auditory stimuli in each of these subregions (Figures 8D–F), suggesting that this measure can reveal differences locally within the IC. These results support the view that despite the lack of plentiful and powerful overt spiking responses in the tonotopic IC, visual information may potentially reach this subregion.

To summarize, the sample along tonotopic penetrations is limited, and spiking responses at these sites tend to be subtle. This makes it difficult to be sure whether there are indeed signals that can reasonably be referred to as “visual” along these penetrations. The available evidence suggests that, if present, visual signals are sparser, weaker, and less temporally coupled to the onset of the visual stimulus in the tonotopic part (putatively the central nucleus) than in other regions of the IC. Nonetheless, it is clear that visually sensitive cells are not confined to a single region in the tissue surrounding the central nucleus.

### Spatial sensitivity

Though a variety of visual receptive field shapes and sizes among IC neurons were identified in our previous study (Porter et al., 2007), population analysis was previously precluded by a data set that was not collected from locations distributed throughout the structure. To summarize spatial receptive field characteristics, we fit two-dimensional Gaussian surfaces to the activity in each of the epochs used for assessing visual responses based on the eye-centered location of the stimulus (see Materials and Methods). We allowed both positive (i.e., peaks) and negative amplitude (i.e., troughs) surfaces. A negative amplitude function was not necessarily indicative of an inhibitory response, the spatial sensitivity was simply better characterized by a circumscribed lower-response region than a circumscribed higher-response region.

**Figure 9:**
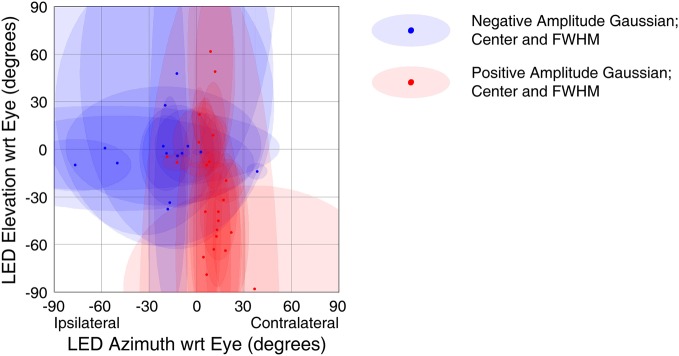
**Spatial tuning of visual responses.** Receptive fields across all visually sensitive sites were summarized using a Gaussian fitting procedure (see Materials and Methods). The centers of the Gaussian functions are shown with red (Gaussian peaks) and blue (Gaussian troughs) points, and ellipses showing the width in the horizontal and vertical dimensions at half the maximum height of the Gaussian (with respect to its offset). Visually responsive recordings reliably showed increased firing rates to stimuli presented in the contralateral hemifield.

The strongest trends were seen when using a response window 50–250 ms window following stimulus onset (this result is predicted as this window was chosen to capture extrafoveal visual sensitivity). Figure 9 shows the receptive field locations of all sites that both showed a significant response in this window (*t*-test, *p* < 0.05) and were successfully fit by a Gaussian function (*f*-test, *p* < 0.05). Of 71 sites meeting the first of these criteria, 29 sites (~41%) met both. Red points in Figure 9 indicate the locations of centers of positive amplitude Gaussian fits, and blue points indicate locations of centers of negative amplitude Gaussian fits. Ellipses show the full width half maximum in the horizontal and vertical dimensions, indicating the size and shape of the receptive field. Positive amplitude Gaussian functions dominate the right hand side of the figure, and negative amplitude functions dominate the left. These results show that, across the population, recordings were more likely to show excitatory visual responses when the stimulus was in the contralateral hemifield. Indeed, points in Figure 9 that do not fit the trend show very small receptive fields, perhaps indicative of spurious receptive field identification. An alternative analysis using multivariate regressions to characterize spatial tuning produced comparable results: slopes of the horizontal component of regressions were biased in favor of increased activity when the stimulus was in the contralateral hemifield (data not shown). The design of our study did not allow for establishing the frame of reference of these response patterns. However, if visual information were strongly and uniformly head-centered, the eye-centered analysis employed here would have been handicapped at revealing spatial sensitivity.

## Discussion

We presently report that visual responses in the IC are not confined to a single subregion: they are well spread in the structure. In our most peripheral recordings, along penetrations that showed little to no evidence of tuning to sound frequency, the majority of sites were visually sensitive (38/47 sites). In recordings along penetrations that showed tuning to sounds, but no evidence of a tonotopic progression, we found fewer visual-related responses (35/110 sites). Along tonotopic penetrations, those that likely passed through the central nucleus, a connection was seen between visual stimuli and neural response (11/42 sites), but it involved notably weaker responses as seen in Figures 7, 8.

The fact that visually responsive neurons are not confined to a small subregion within the IC has important implications. Somatosensory inputs to the IC in the cat are restricted. Their presence has been identified both anatomically and physiologically in the lateral nucleus of the structure but not elsewhere (Aitkin et al., 1978, 1981). Interestingly, the same lateral region has been highlighted in research in owls as a putative site of audiovisual integration (Feldman and Knudsen, 1997; DeBello et al., 2001). Auditory responses of neurons in the owl lateral nucleus can be modulated by a visual stimulus (Bergan and Knudsen, 2009), and exhibit overt visual responses when inhibitory signals from the optic tectum are blocked pharmacologically (Gutfreund et al., 2002). Coupled with results in the somatosensory domain, this suggests the lateral nucleus may have a specialized role in multisensory integration: computations underlying the combination of the senses might be handled by a discrete set of circuits in a localized part of the IC.

Our findings offer partial support for but also refinement of this view. In primates, the effects of visual stimuli are spread through the IC, but are clearly stronger in the regions showing untuned or tuned but not tonotopic auditory responses. We found only minimal sensitivity to visual stimuli along tonotopic penetrations. This suggests that visual signals in the central nucleus, the principal ascending auditory region of the IC, are, if indeed present, weaker/sparser. The IC is a three dimensional structure, so recording penetrations through the central nucleus likely passed through shell regions as well. Indeed, it is possible that the small visual signals we did observe along tonotopic penetrations may have come from the shell locations before or after the tonotopic domain. Additional sampling with finer spacing along tonotopic penetrations is necessary to confirm that visual responses are found at above chance levels within this region. Thus, compelling proof of whether or not neurons in the central nucleus show modulation based on visual signals is still needed. In any event, it is now apparent that the strength and prevalence of visual influences is certainly reduced in the tonotopic area relative to surrounding tissue. Subthreshold evoked potentials, however, were fairly similar across subregions of the IC. These results indicate the potential for visual *modulation* of auditory activity in all regions of the IC when stimuli are presented concurrently. Indeed, auditory cortex has shown LFP changes to non-auditory stimuli in the absence of changes in firing rate (Lakatos et al., 2007). This input can modulate bimodal spiking activity without having a unimodal effect, a pattern also seen in the owl IC (Bergan and Knudsen, 2009).

Anatomical studies have revealed multiple visually sensitive regions that project to shell regions of the IC, including connections from visual and parietal cortices (Cooper and Young, 1976; Coleman and Clerici, 1987; Druga et al., 1997), the retina (Itaya and Van Hoesen, 1982; Yamauchi and Yamadori, 1982; Herbin et al., 1994), and the SC (Adams, 1980; Coleman and Clerici, 1987; Covey et al., 1987). Direct inputs to the central nucleus from visual areas have not been conclusively demonstrated, though such information could potentially reach the IC through less direct routes. The weak visual modulation we observed along tonotopic penetrations might have arisen through such connections. Future studies with concentrated sampling in the central nucleus will be able to conclusively indicate whether centrally located neurons are sensitive visual input.

Visual information may also reach the IC through descending connections within the auditory pathway. A number of papers have reported both modulation and overt effects of visual stimuli in the core and belt regions of auditory cortex [humans: (Sams et al., 1991; Calvert et al., 1999; Giard and Peronnet, 1999; Callan et al., 2003; Wright et al., 2003; Besle et al., 2004, 2008; Molholm et al., 2004; van Wassenhove et al., 2005; Martuzzi et al., 2007; Luo et al., 2010); monkeys: (Barraclough et al., 2005; Brosch et al., 2005; Ghazanfar et al., 2005; Kayser et al., 2007, 2008, 2010); ferrets: (Bizley et al., 2007; Bizley and King, 2008); reviews: (Calvert, 2001; Ghazanfar and Schroeder, 2006; Kayser et al., 2009); appendix]. Descending connections from the auditory cortex more heavily target the shell regions of the IC than the central nucleus (Diamond et al., 1969; Andersen et al., 1980; Druga and Syka, 1984a,b; Coleman and Clerici, 1987; Saldaña et al., 1996; Druga et al., 1997; Winer et al., 2002; Schofield, 2009), consistent with the response trend we observed.

We also conducted the first population analysis of spatial sensitivity to visual stimuli in the IC. We found that spatial tuning was powerfully lateralized with the largest increases in firing rate coming from stimuli in the contralateral hemifield. Auditory responses show similar spatial sensitivity: IC neurons show monotonoic sensitivity with increasing response amplitude as stimuli are presented farther contralaterally (Groh et al., 2003). Further study on single unit responses will be necessary to determine whether individual cells show a relationship between visual and auditory tuning, but evidence from the population suggests that there may be a link.

The contralateral pattern of visual receptive fields parallels that observed for eye position, where firing rates increase as the eyes are oriented more contralaterally (Bulkin and Groh, 2012) However, the two effects are distinct, and neither can fully account for the other. For example, if the IC were not actually sensitive to visual stimulus location but only to eye position, this would appear in our study as a preference for visual stimulus locations in the opposite hemifield. When the eyes are directed more contralaterally, evoking higher eye-position related activity, the location of the fixation LED would lie more ipsilaterally on the retina, and neurons would appear to have ipsilateral visual receptive fields. We did not observe this pattern, suggesting that eye position and visual signals influence IC neurons independently.

These results raise questions about how auditory, visual, and eye-position signals interact in the IC. To combine the senses, the brain must reconcile signals that arise in different frames of reference: auditory localization is anchored to the head, and visual localization is anchored to the (independently moveable) eyes. In our assessment of spatial sensitivity we used an eye-centered definition of visual location, but the coordinate system used by the brain may be different. On average, auditory information in the IC is not encoded in head-centered frame of reference (as might be assumed given the head centered methods for assessing sound location). Rather auditory response patterns are modulated by the position of the eyes, though not enough to transform them fully into eye-centered co-ordinates (Groh et al., 2001; Zwiers et al., 2004; Porter et al., 2007; Maier and Groh, 2010). Visual signals may show a similar pattern, with receptive fields that are not fully described in a retinotopic reference frame. Such results have been observed in lateral and medial intraparietal cortex, representations of visual and acoustic information span a continuum from head- to eye-centered (Mullette-Gillman et al., 2005, 2009).

Along tonotopically organized penetrations through the IC, putatively the central nucleus, overt visual effects are weak at best while eye position modulation is still quite strong (Bulkin and Groh, 2012). While responses to visual stimuli are weak in the central nucleus, the effect of visual cues during bimodal stimulus presentation remains untested. Visual input may reach the central nucleus, but be ineffective in changing spike rate in the absence of auditory stimuli, a pattern similar observed with somatosensory effects in auditory cortex (Lakatos et al., 2007). Future work using presentations of bimodal stimuli will investigate this possibility.

### Conflict of interest statement

The authors declare that the research was conducted in the absence of any commercial or financial relationships that could be construed as a potential conflict of interest.
